# Human iPSC‐derived neuron modeling for the study of early‐onset Alzheimer’s disease

**DOI:** 10.1002/alz.094681

**Published:** 2025-01-09

**Authors:** Phoebe Valdes, Andrew B. Caldwell, Qing Liu, Kenneth W. Henry, Michael Q Fitzgerald, Koushik Muralidharan, Srinivasan Ramachandran, Celeste M. Karch, Dominantly Inherited Alzheimer Network (DIAN), Shauna H Yuan, Steven L Wagner, Lawrence S.B. Goldstein, William C. Mobley, Douglas R. Galasko, Shankar Subramaniam

**Affiliations:** ^1^ Department of Bioengineering, University of California, San Diego, La Jolla, CA USA; ^2^ Department of Neurosciences, University of California, San Diego, La Jolla, CA USA; ^3^ Department of Cellular and Molecular Medicine, University of California, San Diego, La Jolla, CA USA; ^4^ School of Medicine, University of California, San Diego, La Jolla, CA USA; ^5^ Department of Psychiatry, Washington University in St. Louis School of Medicine, St. Louis, MO USA; ^6^ Department of Neurology, University of Minnesota, Minneapolis, MN USA; ^7^ Department of Neurosciences, University of California San Diego, La Jolla, CA USA

## Abstract

**Background:**

Early‐onset Alzheimer’s disease (EOAD) is a complex disease that occurs at an early age at onset (AAO) before 65 years, constituting 5‐6% of all AD cases and remains poorly understood. Patient‐derived induced pluripotent stem cells (iPSCs) have been used to model different forms of EOAD that display heterogeneous disease mechanisms.

**Method:**

We examined iPSC‐derived neurons from both familial EOAD harboring mutations in *PSEN1^A79V^*, *PSEN2^N141I^, and APP^V717I^* and non‐familial EOAD patients at an early AAO. RNA‐seq for familial and non‐familial EOAD patients as well as ATAC‐seq for familial EOAD patients were carried out to characterize the gene expression and chromatin accessibility changes, respectively. Differential expression and enrichment analysis, TF activity identification, and co‐expression module detection were performed for familial EOAD RNA‐seq. Clustering and surrogate neuron marker classification were performed for non‐familial EOAD RNA‐seq. Differential peak analysis, TF motif footprinting and peak functional enrichment were performed for familial EOAD ATAC‐seq.

**Result:**

Our approach allowed us to identify the correlation between gene expression and chromatin accessibility associated with key disease familial EOAD endotypes. We identified limitations with our non‐familial EOAD neuron model to study sporadic AD, providing evidence that these neurons present variation of differentiation across patient clones, patient variability and an immature culture state. Common endotypes were identified across three familial EOAD mutations such as dedifferentiation of a mature neuron to a less differentiated quasi‐neuron state and repression of mitochondrial function and metabolism. Integrative analysis allowed us to ascertain the master transcriptional regulators associated with these endotypes, including REST, ASCL1, and ZIC family members (activation), as well as NRF1 (repression). Our non‐familial EOAD study showed a modest difference in expression profiling and a limited number of differentially expressed genes (DEGs) between diseased and control subjects.

**Conclusion:**

iPSC‐derived neurons demonstrated that familial EOAD mutations share common regulatory changes within endotypes with varying severity, leading to reversion to a less‐differentiated neuron state. Extending the usage of these neurons to non‐familial EOAD may not serve as ideal to study sporadic AD. Overall, we have demonstrated that human neuron modeling can be applied to different forms of EOAD to understand the disease etiology better.

